# Circadian Rhythms in Fear Conditioning: An Overview of Behavioral, Brain System, and Molecular Interactions

**DOI:** 10.1155/2017/3750307

**Published:** 2017-06-18

**Authors:** Anne Albrecht, Oliver Stork

**Affiliations:** ^1^Department of Genetics & Molecular Neurobiology, Institute of Biology, Otto von Guericke University of Magdeburg, Leipziger Straße, 4439120 Magdeburg, Germany; ^2^Center for Behavioral Brain Research, Magdeburg, Germany

## Abstract

The formation of fear memories is a powerful and highly evolutionary conserved mechanism that serves the behavioral adaptation to environmental threats. Accordingly, classical fear conditioning paradigms have been employed to investigate fundamental molecular processes of memory formation. Evidence suggests that a circadian regulation mechanism allows for a timestamping of such fear memories and controlling memory salience during both their acquisition and their modification after retrieval. These mechanisms include an expression of molecular clocks in neurons of the amygdala, hippocampus, and medial prefrontal cortex and their tight interaction with the intracellular signaling pathways that mediate neural plasticity and information storage. The cellular activities are coordinated across different brain regions and neural circuits through the release of glucocorticoids and neuromodulators such as acetylcholine, which integrate circadian and memory-related activation. Disturbance of this interplay by circadian phase shifts or traumatic experience appears to be an important factor in the development of stress-related psychopathology, considering these circadian components are of critical importance for optimizing therapeutic approaches to these disorders.

## 1. Introduction

Oscillation of behavioral and physiological processes over the course of the day is observed across the animal kingdom and provides an evolutionary conserved feature to anticipate and settle for basic needs such as food and resting periods [1]. In mammals, circadian rhythms are paced by a small nucleus, the suprachiasmatic nucleus (SCN), within the hypothalamus [2, 3]. Timing of the master clock is achieved by transcriptional/translational feedback loops that drive rhythmic changes in the expression of specific clock genes. In brief, the feedback loop contains the clock proteins CLOCK and BMAL1, which form an activator complex that functions as a positive transcription factor for the period (PER) and cryptochrome (CRY) genes. The PER and CRY proteins in turn form heterodimers that translocate to the nucleus and function there as a repressor for the CLOCK:BMAL1 complex. CRY:PER complexes are degraded over time in ubiquitin-dependent pathways to a sufficient level to relieve the repression on CLOCK:BMAL1, thus generating an approximately 24-hour cycle. Further, kinases control the nuclear shuttling and degradation of the CRY:PER complex to adjust it to external stimuli [4–6]. Such a cellular clock is especially well coupled and synchronized within neurons of the SCN, but it is found in other brain regions as well as in peripheral tissues. In the latter, they drive circadian expression of genes involved in a wide range of physiological processes such as metabolism, sleep/activity, and control of body temperature, and these cues can feedback to the SCN in order to adjust the internal clock [2, 3, 7, 8]. Accordingly, it can be assumed that circadian rhythms in brain regions other than the SCN help to coordinate different aspects of sensorimotor coordination and higher brain functions with a circadian rhythm.

Over the past decade, evidence has accumulated that the circadian system modulates fundamental processes of learning and memory formation [9, 10]. Forming episodic memories, that is, acquiring and remembering information about previous events, is especially important for an individual and crucial for promoting its survival and well-being. Anchoring such memories to day and activity phases can be useful to predict similar events in the future. These functions are particularly relevant as it comes to the experience of aversive events and the development of coping or avoidance mechanisms upon learning. Classical “Pavlovian” fear conditioning is a powerful and evolutionary well-preserved process observed across various species serving this function [11]. In this review, we will discuss the current state of knowledge concerning the interaction of the fear memory and circadian system at the behavioral, molecular, and brain systems levels.

## 2. Procedures, Processes, and Circuits of Fear Conditioning

Pavlovian fear conditioning is frequently employed by researchers to study the fundamental neurobiological processes of aversive memory formation. Here, an innately aversive event, the so-called unconditioned stimulus (US), is coupled to an initially neutral stimulus, which thus becomes the conditioned stimulus (CS). Rodents are the most widely used model organisms to study fear conditioning. Here, mild electrical footshocks as the US are often presented together with tones as the CS during the conditioning phase. Consequently, the CS tone will develop predictive value for the US [12]. The strength of the fear memory is tested during the retrieval phase, usually 24–48 h after training to test for long-term memory or even weeks later to test for remote fear memory. During retrieval, the animal is re-exposed to the tone and its defensive behavior comprising of freezing (immobility except for respiratory movement), and risk assessment (orientation towards the stimulus, alert watching with head movements, and stretched attend posture) is monitored [13]. The amount of freezing is directly related to the strength of the fear memory trace, with less intensive training (few US-CS pairings, low footshock intensity) resulting in low freezing and intensive training (higher number of CS-US pairings and high footshock intensity) in higher levels of freezing [14, 15].

Cues with other modalities can function as a CS as well (e.g., a light stimulus or an olfactory stimulus), but also the complex, multimodal environment in which the foot shock is delivered can elicit a fear response. Such contextual fear conditioning takes place in the absence of a discrete CS [16], but to some degree, also during any stimulus-dependent fear conditioning procedure. But since the cue is the more salient fear-eliciting stimulus, the context of the initial CS-US pairing is then referred to as the background context [17].

Importantly, fear memory formation is not a singular event, but, once formed, memory traces can undergo constant modification. During retrieval of conditioned fear, the original fear memory trace becomes labile and susceptible to modulation. While in the absence of an interfering stimulus, the fear memory trace is maintained; it can also be updated with new information, a process called reconsolidation [18, 19]. Reconsolidation thereby requires similar biochemical processes as consolidation of the original fear memory [20, 21]. However, if the CS is repeatedly reintroduced to the animal without reinforcement by concomitant US pairings, the fear memory trace towards the CS is extinguished. During extinction training, a new memory trace is formed and the individual learns that the CS should no longer elicit a fear response and the original fear memory trace is suppressed [22].

For the acquisition and consolidation of a fear memory trace, its retrieval, and its extinction, three key brain regions have been in the focus of attention: the amygdala, hippocampus, and prefrontal cortex. During fear conditioning, sensory information from various thalamic and cortical areas as well as nociceptive information are conveyed to the lateral subnucleus of the amygdala (LA), thus providing the neurobiological basis for CS-US pairings [23]. The LA then provides inputs into the central nucleus (CeA), which projects to various areas in the brain stem, hypothalamus, and periaqueductal grey that generate the actual fear response consisting of freezing, peripheral arousal, and stress hormone release [13]. The LA is further closely connected to the basolateral nucleus (BLA) and both are often referred together as the basolateral complex. The BLA is believed to receive contextual information from the hippocampus [24]. Indeed, many lesion studies demonstrate that the hippocampal formation is required for establishing contextual and traced fear memory (with a temporal separation of CS and US) [16, 25, 26]. The dentate gyrus (DG) subregion of the hippocampal formation receives input from the entorhinal cortex via the perforant pathway. Information are then projected to the Cornu ammonis subfield (CA3) via mossy fibers and from there to CA1 via Schaffer collaterals but are also closely interconnected and allow for example for backpropagations of the CA3 to DG [27, 28]. Within this so-called trisynaptic pathway, spatial and temporal information are processed before being projected to the BLA and other brain areas. However, in reverse, the BLA is also able to modulate plasticity in the dentate gyrus and CA1 areas [29], and LA and dorsal CA1 synchronize their oscillatory neuronal activity at the theta frequency range during encoding and retrieval of cued and contextual fear memory [30, 31]. Of note, direct anatomical projections from the BLA exist to the ventral pole of the hippocampal formation [32]. Indeed, many lesion studies as well as pharmacological and optogenetic interventions demonstrate that especially the ventral hippocampus contributes to the formation and expression of cued and contextual fear memory, while the dorsal portion mediates predominantly cognitive processes such as spatial learning and pattern separation processes [33–36]. However, cognitive processes in the dorsal hippocampus might also be required for determining the specific context in which fear conditioning took place, thereby avoiding generalization of fear memory to other nonreinforced stimuli or environments [37–40].

The medial prefrontal cortex is indispensable for extinction of fear memory. The infralimbic portion of the PFC (IL) sends strong projections to inhibitory cells located between the BLA and CeA regions of the amygdala, the so-called intercalated (ITC) cells. During extinction memory retrieval, these ITC cells are activated via IL inputs and inhibit the output from the CeA, thus preventing a fear response. The prelimbic portion of the mPFC (PL), however, sends excitatory projections to the BLA and is active during fear conditioning itself, leading to an increased fear response [22]. The mPFC also tightly controls activity in the hippocampal formation. Correspondingly, fear extinction is highly context specific and lesions of the hippocampus interfere with extinction memory [41, 42]. Together, a bidirectional circuit between the amygdala, hippocampus, and PFC mediates fear memory consolidation, retrieval, and extinction.

In order to establish a long-term fear and extinction memory, consolidation processes take place in all of the involved areas, which lastingly facilitate the interplay of such neurons that have been activated during acquisition. This is achieved on a cellular level by a reorganization of the synapse, including rearrangement of neurotransmitter receptors and other synaptic proteins as well as modulation of extracellular matrix proteins around the synapse [43]. These processes are dependent on transcription of specific target genes and translation into proteins, which are initiated via intracellular signaling cascades. During both fear and extinction memory formations, activation of neurons leads to an increase in intracellular calcium levels, which activate the second messenger protein cyclic adenosine monophosphate (cAMP) that in turn triggers the activation of protein kinases such as protein kinase A (PKA) and the extracellular-regulated kinase (ERK)/mitogen-activated protein kinase (MAPK). Activated MAPK then enters the nucleus and phosphorylates the cAMP response element-binding protein (CREB), which serves as a transcription factor [21, 44]. Together with other transcription factors, for example c-Fos or c-Jun, CREB mediates fear conditioning-dependent expression changes in numerous target genes in a highly complex pattern [45–47].

## 3. Fear Conditioning and Extinction: Timing Matters

Mice and rats, the most commonly used model organisms in fear memory research, are nocturnal animals and display their highest activity during the dark phase [48–50].

Several studies investigated whether fear memory strength depends on the circadian phase. In mice, acquisition of cued fear memory is faster when training takes place in the inactive light phase, and freezing levels are higher when tested during the inactive phase, regardless of the initial training time point [51]. Increased fear memory to a conditioned tone is also observed in rats tested and trained in the inactive, light phase as compared to those in their active phase [52]. A similar relationship has previously been reported for amygdala-dependent conditioned taste aversion, where memory strength is greater when conditioning occurs during the inactive phase [53]. Another study performed in rats found that using a less-intensive auditory-cued fear conditioning protocol than the one used in [52] found no differences on acquisition and retrieval of conditioned fear when comparing training and retrieval during the light versus the dark phase [54]. However, this study reported a phase-dependent effect on extinction learning, which is facilitated when extinction training and testing are performed during the active, dark phase [54]. This is in agreement with findings in mice that showed increased extinction rates when extinction training takes place during the dark phase [51].

Modulating the time point of training and testing within the dark or light phase (e.g., the beginning of the dark phase versus second half of the dark phase) has no apparent impact on fear memory strength in mice and rats [50, 55, 56] but affects extinction in humans. In young men, extinction learning is improved in the morning compared to that in the evening and correlates with higher levels of the stress hormone cortisol in the morning [57]. The facilitation of extinction during the inactive phase in rats is also dependent on natural fluctuations of glucocorticoids and abolished by adrenalectomy.

Furthermore, rhythmical patterns have been observed for the retrieval of aversive memories. As initially described by Kamin in 1957, optimal performance in an active avoidance tasks occurs at 24 h intervals between training and testing [58–60]. Further experiments revealed that retrieval of avoidance memory is most successful at periodic 24 h intervals between training and testing (e.g., 24, 48, and 72 h after training), while impairments that occur in those intervals are shifted for 6 h (e.g., retrieval of 6 h, 18 h, and 30 h after training) [61, 62]. This suggests circadian state dependency of aversive memory retrieval or “timestamping”. A similar effect is observed in contextual fear conditioning, where fear memory retrieval is enhanced when the testing is performed at 24 h intervals after training [63, 64]. In this way, the time of the training becomes part of the training context and re-exposure to the time component facilitates retrieval. Similar effects are not observed for the more salient cue [12, 50, 51, 56]. However, in animals trained with auditory cues, timestamping is also observed for background context fear memory, as contextual retrieval is impaired at 12 h but not 24 h after training [65]. Interestingly, timestamping of the background context is only effective when the training occurs during the light, inactive phase [65], when contextual fear memory formation is enhanced [51].

Interestingly, when contextual and auditory-cued fear conditioning trainings and testing are performed in the inactive phase and posttraining sleep restriction is added to the protocol, the freezing levels are rather reduced [52, 66]. By contrast, fear memory obtained in the dark phase appears much more resistant to the effects of sleep deprivation [66], thus pointing towards the importance of sleep for memory consolidation processes that might be also relevant for the circadian effects in fear memory formation [67]. Indeed, postacquisition processes last well into sleep phases, and neuronal activity of the amygdala, hippocampus, and prefrontal cortex during rapid-eye-movement (REM) sleep appears to support the consolidation of emotional memories [68, 69]. Accordingly, inhibiting oscillatory brain activity in the hippocampus during REM sleep phases within a 4 h time window after cued fear conditioning disrupts contextual fear memory components [70]. In accordance with the general circadian activity pattern, sleep deprivation has a more pronounced impact on fear memory formation when performed during the inactive phase, where sleep phases are more likely [66]. However, for the retrieval of extinction memory, the circadian phase of sleep deprivation seems less important and does not correlate with the sleeping time [71]. Thus, extinction memory efficacy may relate to a greater extent to sleep-specific neuronal events, while additional circadian mechanisms may contribute to the consolidation of the initial fear memory trace.

## 4. Disrupting Fear Memory by Circadian Shifts

In addition to the observed mild impact of the circadian time of testing on fear and extinction memory formation, disruptions of the circadian rhythm can indeed have negative consequences on emotional memory formation. These findings are of special relevance, since acute circadian phase shifts in forms of jet lag and shift work are increasing in modern life.

In mice, acute phase shifts of six hours or more in the light-dark cycle reduce contextual fear memory, in parallel to increased glucocorticoid release and disruption of sleep patterns [64]. Reduced cued fear memory has been observed as well in “jet-lag model”, consisting of six 8 h advanced light shifts within 18 days [65]. However, applying such photoshift protocols repeatedly (4 sessions with phase advances for 3 h over 6 days, 64 days protocol in total) alters hippocampus-dependent spatial learning but leaves cued and contextual fear conditioning unaltered or even increases it [72–74].

As reviewed by Krishnan and Lyons [9], disruption of circadian rhythms affects not only fear memory formation but also other learning and memory paradigms. For example, shortening of the light-dark cycle to 11 h-11 h phases dissociates locomotor activity rhythms in rats and impairs passive avoidance learning while leaving more cognitive tasks such as object recognition memory formation unaltered [75]. In Siberian hamsters made arrhythmic by a brief light pulse paradigm, cognitive problems arise with deficits in novel object recognition memory and spontaneous alternations [76, 77]. Interestingly, lesion of the SCN rescues these cognitive deficits [78].

In reverse, circadian shifts can be also induced through aversive events. Social defeat stress induced by interactions with other aggressive animals, for example, alters circadian undulations of locomotor activity, body temperature, and heart rate without affecting internal circadian rhythms measured under free-running dark conditions (see [48] for review). This suggests that while the SCN master clock works unaltered by social stress, subordinate oscillators are affected. However, severe stress, such as recurrent foot shocks on specific day times, is able to shift behavioral activity rhythms even under free-running conditions and is dependent on the function of the SCN in conjunction with the amygdala [79]. This further illustrates that the regions important for fear memory can coordinate the circadian rhythm of behavior in response to a fear-evoking threat, working as subordinate circadian oscillators.

## 5. Clock Genes Are Expressed in Fear Memory Brain Circuits

How may the amygdala, hippocampus, and PFC function as subordinate circadian oscillators? Clock genes are expressed in these regions and show diurnal expression patterns. PER2 is the best investigated example, with the highest expression levels in the BLA and in the dentate gyrus and CA13 subareas of the hippocampus towards the end of the dark phase and at the beginning of the light phase, while expression levels in the CeA and in frontal cortical areas peak at the beginning and the middle of the dark phase [50, 80–82]. In other clock genes such as PER1, CRY1 and CRY2, CLOCK and BMAL1 show diurnal expression patterns, at least in the hippocampal formation, as well [83]. Lesion of the SCN reduces the oscillation of PER2 expression in the amygdala subnuclei and the DG [80], suggesting that the SCN master clock drives limbic oscillations of PER2. Noteworthy, glucocorticoids, which are released with a circadian pattern from the adrenal glands under baseline conditions and as a bolus under stress, can modulate clock gene expression levels as well. Adrenalectomy abolishes PER2 expression rhythms only in the CeA [80] and in frontal areas [82] but not in the BLA and DG [80] or in the SCN itself [82]. In the CeA and frontal areas, PER2 rhythms can also be modulated by application of external glucocorticoids [82, 84]. For the CeA, it was further demonstrated that knock down of the HPA axis peptide CRF as well as chronic stress exposure induces shifts in rhythmical PER2 expression, while mice lacking the glucocorticoid receptor display no PER2 oscillations (see [84] for review). An acute stressor such as exposure to a fear-inducing odor found in the feces of predator animals is able to shift oscillatory rhythms of PER2 expression not only in the CeA but also in the hippocampus and the BLA [85].

Together, these findings suggest that clock gene expression oscillations may be shifted by stressful stimuli in brain regions relevant for fear memory and its extinction. Whether clock genes themselves are crucial for fear memory formation may be determined using transgenic mice, but results so far are not fully conclusive. Mice lacking the PER2 gene display decreased hippocampus-dependent trace fear memory in which they have to remember a time contingency between a CS and US. By contrast, simple auditory-cued fear memory is unaffected in these mutants [81]. In another study however, hippocampus-dependent spatial memory as well as contextual fear memory were found unaffected in PER2 and also PER1 knock out mice [86]. Mutant mice carrying a double knock out for the PER2 binding partners CRY1 and CRY2 likewise display no deficits in cued memory [87]. Chryptochromes, however, inhibit a complex of the clock proteins CLOCK and BMAL1, which function as transcriptional activators in the circadian expression feedback loop of clock genes [4]. When BMAL1 is abolished, such transgenic mice show impaired spatial and contextual fear memory [88].

Interestingly, BMAL1 and also PER2 knock out mice display perturbed MAPK pathway activation in the hippocampus [81, 88], suggesting that memory deficits observed in these animals may involve impairments in key molecular pathways of fear conditioning.

## 6. Molecular Rhythms of Fear Conditioning

Reduced levels of phosphorylated CREB and a lack of circadian oscillations have been observed in PER2 and also in PER1 knock out mice [81, 89]. Thus, activation of CREB as a transcription factor happens with a circadian pattern and appears to be driven by clock genes. Indeed, variations in spatial memory strength are dependent on the daytime of testing and may relate to circadian rhythms of CREB phosphorylation [89]. A possible mechanism for such gating effects of clock genes on the MAPK pathway has been recently described by Rawashdeh et al. [90]. In their model, PER1 is suggested to form a complex with the MAPK-activated ribosomal S6 kinase (p90RSK), which translocates to the nucleus upon neuronal activation. There, p90RSK is a potent modulator for CREB phosphorylation. Together, this can explain why activation of the MAPK pathway, facilitating hippocampus-dependent spatial memory formation, becomes especially effective in stimulating CREB phosphorylation when expression levels of PER1 are high during the light phase. This matches the circadian pattern of contextual fear memory strength, inducing the highest freezing levels when both fear conditioning training and testing are performed during the light phase [90]. The behavioral time pattern coincides with a peak in activation of MAPK pathway components upstream of P-CREB during the light phase, namely, the second messenger cAMP and the phosphorylation forms of the kinases MEK1/2 and ERK1/2 [91]. This circadian oscillation of MAPK pathway activation is impaired after lesion of the SCN [92], suggesting modulation by the superordinate circadian rhythm generator. Similar to activation of their upstream kinases of the MAPK pathway, the eukaryotic translation initiation factor 4E (eIF4E) and the eIF4E-binding protein (4EBP) display a peak in phosphorylation during the light phase and are significantly reduced during the dark phase. Abolishing circadian rhythms by continuous light exposure (12 h/12 h light/light cycle) disrupts the circadian phosphorylation pattern of the translation initiation factors and reduces contextual fear memory strength [93]. Noteworthy, the peaks in activation of memory-related MAPK signaling cascades as well as protein translation occur during the inactive phase and might relate to system consolidation of memory during sleep. Accordingly, inhibition of translation during the inactive phase two days after training, when the memory trace has been already established, impairs remote retrieval of fear memory [93].

While these processes are well described for hippocampal neurons, it remains to be resolved whether similar processes take place in other areas, such as the amygdala and frontal cortical areas, and may add to circadian effects on cued fear conditioning and extinction.

## 7. Circadian Rhythms in Neuromodulator Release

Coordinating the activation of multiple brain regions during fear memory formation requires long-distance network interactions and the adjustment of activity through release of neuromodulators. During an aversive task such as fear conditioning, the glucocorticoid stress hormones and the monoaminergic neurotransmitter noradrenaline are strongly released, supporting memory consolidation [94]. Circadian rhythms of glucocorticoid release are well described in rodents and humans. Plasma concentrations of corticosterone, the main glucocorticoid in rodents, are maximal at the beginning of the dark phase and minimal in the morning after the onset of the light phase [95]. Such a circadian rhythm of corticosterone release is driven by SCN inputs to its neighboring structure within the hypothalamus, the paraventricular nucleus (PVN) [96]. The PVN regulates the release of hormones that lead to glucocorticoid release from the adrenal glands (hypothalamic-pituitary-adrenal axis, HPA axis) [97]. The glucocorticoid receptor (GR) itself is a powerful transcription factor that also targets the clock genes PER1 and PER2 and can induce phase shifts of circadian rhythms in subordinate oscillators. In addition, the PER-responsive CLOCK/BMAL1 complex can regulate transcriptional activity of GRs via epigenetic mechanism [96]. Thus, the HPA axis for mediating stress responses and the circadian clock system are closely interacting on multiple levels. Boosting corticosterone levels shortly before fear conditioning training facilitates fear memory consolidation through activation of GR [98–100] and is required for establishing extinction memory [101, 102]. Upon retrieval, high levels of corticosterone can also interfere with reconsolidation processes, thereby weakening the original fear memory [103, 104]. Accordingly, in mice with high levels of endogenous corticosterone plasma levels during retrieval of an auditory-cued fear, increased freezing to the background context is observed upon a second retrieval [105]. This suggests that high levels of corticosterone can induce a form of generalization by reconsolidation processes. Thereby, the specificity of the fear memory is reduced and the conditioned response generalizes to cues with less predictive values. Moreover, a detailed analysis comparing animals with high or low corticosterone responses of the HPA axis during different phases of the dark cycle (the beginning of dark cycle with high plasma corticosterone levels versus the middle of the dark phase where corticosterone plasma levels are already lowered) showed that the generalization effect is not observed when reactivation takes place during the beginning of the dark phase, that is, in those animals with a blunted combined circadian and stress-induced corticosterone response during reconsolidation [105]. The loss of the predictive specificity of cue and context in cued versus unpaired, contextual fear conditioning is also induced by administration of corticosterone during initial fear memory training conducted during the light phase [106], when endogenous corticosterone levels are usually low. Together, these data illustrate that circadian rhythms of the HPA axis and the expression of clock genes interact closely with molecular factors such as MAPK pathway in determining the strength of fear memory. This is also underlined by findings in adrenalectomized rodents, where freezing levels increase in animals trained in the dark phase and daytime effects on extinction are abolished [54]. The data furthermore suggest that an imbalance in this interaction, for example, artificially out-of-phase increase in corticosterone level, leads to a more unspecific fear memory trace. Similar generalization phenomena are observed in patients suffering from posttraumatic stress disorder (PTSD), which can be viewed as maladaptive fear memory formation to the traumatic event [107]. In PTSD patients, altered circadian fluctuation of glucocorticoid levels and hyperresponsiveness of the HPA axis have been reported (see [108, 109] for review). While the first findings on manipulating glucocorticoid levels during retrieval of the traumatic memories seem a promising therapeutic option [110], systematically investigating and utilizing interactions of the circadian clock and HPA axis responsiveness could provide further supportive avenues in those settings.

Pharmacological studies demonstrate that neuromodulators involved in fear memory formation also display circadian rhythms in release and metabolism. Serotonin for example is released from midbrain raphe nucleus neurons that send projections to various brain regions, including those relevant for fear conditioning, as well as to the SCN. Serotonin synthesis and the firing rate of serotonergic neurons are maximal during the inactive phase and are essential for maintaining circadian rhythms of corticosterone release and behaviors such as locomotion and food intake (see [111] for review).

In addition to the possible neuromodulatory regulation of the SCN master clock, circadian rhythms of serotonin levels have been observed within the amygdala and hippocampus, with higher levels towards the end of the dark phase [112]. However, whether serotonin (5HT) augments or impairs fear memory formation also strongly depends its binding to different receptor subtypes. Activation of the 5HT2A receptor subtype in the amygdala results in increased acquisition and expression of cued but not contextual fear memory, while activation of 5HT1A receptors in the amygdala and hippocampus impairs contextual fear conditioning (see [113] for review). Thus, interactions of circadian effects on serotonergic impacts on fear memory strength need further evaluation.

Likewise, acetylcholinergic neurotransmission via certain muscarinergic and nicotinergic receptor subtypes appears to facilitate cued and contextual fear conditioning. It may also regulate the interactions of the amygdalo-hippocampal system together with frontal cortical areas during extinction training (see [114–116] for review). Acetylcholine release is increased in the hippocampus during contextual fear conditioning training and correlates with freezing levels measured briefly after [117]. However, acetylcholine levels also correspond to general locomotor activity and are therefore high during the active, dark phase within the prefrontal cortex and hippocampus [117, 118], whereas the fear memory strength is increased during conditioning and testing in the inactive phase [51, 90]. Performance in a passive avoidance task is more successful during the active dark phase, but it is diminished by blocking the degeneration of acetylcholine. The same pharmacological stimulation has no impact when performed during the light phase, where internal acetylcholine levels are lower. However, when reducing acetylcholinergic neurotransmission further during the light phase, such aversive memory retrieval is impaired while the same blockage has less impact on avoidance memory retrieval during the dark phase [119]. Thus, acetylcholinergic effects on aversive memory strength need to be synchronized with circadian patterns of other factors to be most effective and may contribute, in parallel to the proposed role of corticosterone rhythms, to fear generalization phenomena when out of sync. Accordingly, acetylcholine provides inputs on vasopressin cells of the SCN, thereby providing also a feedback to the master clock. Once activated by acetylcholine, vasopressin in the SCN is repeatedly released with a 24 h delay. Since acetylcholine is especially released during arousing events, as well as during fear conditioning training, such recurrent vasopressin release in the SCN might contribute to the timestamping observed during fear conditioning [63] and might influence phase shifts [120] and the rest of the SCN master clock after fear conditioning [121].

## 8. Summary and Conclusions

Classical fear conditioning is a powerful tool to understand the neurobiological basis of emotional memory formation. Humans display increased fear responses at night [122]; likewise, especially contextual fear conditioning in nocturnal rodents appear to be more effective during their inactive phase, when lights are on.

From an evolutionary perspective, remembering the when and where of a threat through the inactive phase, when the animal is more prone to retreat to and rest in its nest, occurs highly relevant for survival. Encountering a threatening situation during exploration of the environment, foraging, and so on during the active phase however seems more likely; thus, fear memory strength might be tuned down somewhat in order to cope appropriately with expectable aversive events. Thus, circadian effects on fear memory appear linked to the natural activity rhythms of the animals. In this line, disruption of fear memory due to sleep deprivation appears especially effective when performed during the inactive phase, when the probability of sleep is higher.

**Figure 1:**
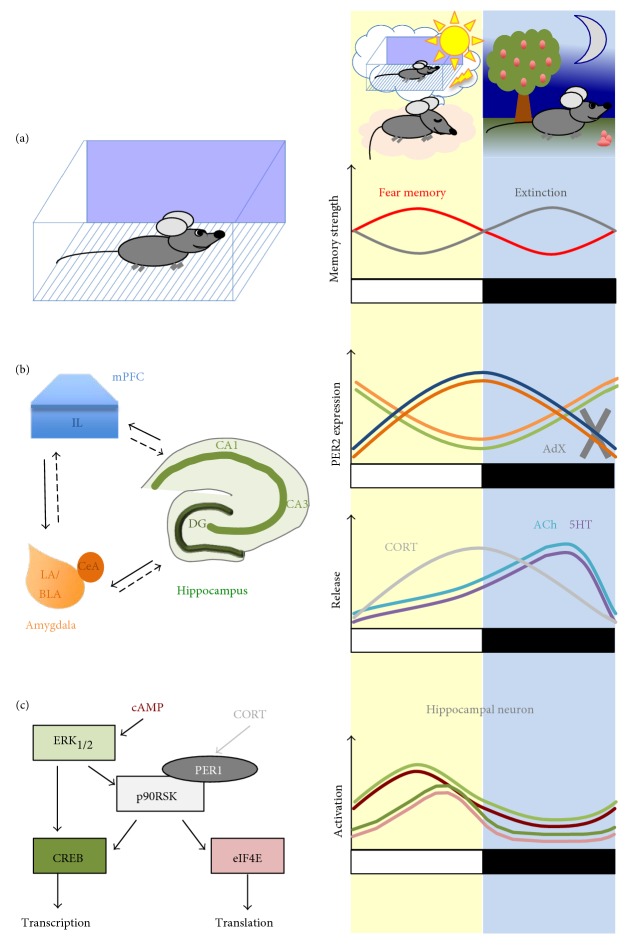
Overview of circadian fear memory system interactions. (a) On the behavioral level, fear memory strength is higher when fear conditioning and its retrieval took place during the inactive, light phase [51], while extinction memory is facilitated when training and testing took place during the dark, active phase [54]. On evolutionary aspects, this may relate to the increased significance of aversive events occurring during the inactive phase, when the animal usually retires to the nest. Further, the increased probability of posttraining sleep, which is required for memory consolidation, may strengthen fear memory acquired during the inactive phase. (b) On the brain circuit level, fear and extinction memory strength are determined by an interaction of the amygdala, hippocampus, and medial prefrontal cortex (mPFC). While cue associations are stored in the lateral and basolateral nuclei of the amygdala (LA/BLA), contextual information is processed by the hippocampal formation (DG: dentate gyrus; CA: Cornu ammonis, 1–3) [13, 16]. For extinction of fear memory, the infralimbic cortex (IL) is important in inhibiting conditioned responses, mediated trough the central amygdala (CeA) [22]. The expression of clock genes such as period (PER) 2 displays a circadian rhythm with different phase settings within the LA/BLA (light orange) and hippocampal subregions (light green) versus the CeA (dark orange) and IL (blue) [80–82]. Adrenalectomy (AdX) abolishes the circadian expression pattern of PER2 only in the CeA and the IL [80, 82], which correspond to circadian rhythms of corticosterone (CORT) plasma levels [95] depicted in the lower graph. In addition, fear memory consolidation strength is modulated by serotonin (5HT) and acetylcholine (ACh), which show circadian variations in amygdala and/or hippocampal tissue levels as well [112, 117]. (c) On the molecular level, it has been shown in hippocampal neurons that different components of the MAPK pathway display a circadian activation profile, with peaks during the light phase for the second messenger cAMP (dark red), the kinase ERK1/2 (light green), the transcription factor CREB (dark green), and the protein translation initiation factor eIF4E (light red) [90, 91, 93]. Moreover, the nuclear translocation of p90RSK, a kinase that activates CREB and modulates translation, is regulated by the clock protein PER1, thereby modulating transcriptional activation of CREB-dependent downstream proteins [90]. PER1 expression levels are further regulated by corticosterone signaling, adding a further level of stress and circadian systems interactions [96].

It is therefore conceivable that the fear conditioning and the circadian system interacts on many levels (see also Figure 1 for summary). First, molecular signaling pathways relevant for the consolidation of long-term fear memories are modulated by genes that drive the internal clock [89, 90], and deficiency in clock genes results in impairments in contextual fear memory [88]. Other learning types addressing hippocampal functions as well as cellular models of learning that lastingly alter synaptic plasticity are similarly modulated by the internal clock (see, e.g., [9, 10] for recent reviews). Whether these processes can also be observed in the amygdala or frontal cortical areas relevant for extinction remains to be investigated in detail. A special role of the hippocampus in combining circadian and contextual processes, however, has become evident, since time-place encoding of an event as part of episodic memory formation is one of the primary hippocampal tasks and most likely would contain also daytime information [63].

Second, neuromodulators such as acetylcholine that display a circadian modulation and may also affect the SCN master clock [120] are crucial for fear memory formation. For the stress hormone corticosterone, the underlying interactions have begun to unravel; they describe circadian effects of reconsolidation and corticosterone levels on fear memory [105] and effects of corticosterone on expression of clock genes in areas relevant for fear memory and extinction [80, 82].

Third, an interaction of circadian rhythms and fear memory are potentially relevant for the pathogenesis and therapy of neuropsychiatric disorders. Excessive fear conditioning and impaired extinction have been implicated as key behavioral mechanisms of anxiety disorders such as phobias, panic disorder, and PTSD. Targeting molecular pathways involved in fear memory consolidation, reconsolidation, and extinction therefore lay the basis for an evidence-based therapy for these disorders [107]. Clock genes, in interaction with modulators such as corticosterone, provide interesting new targets for understanding many neuropsychiatric disorders [123], and understanding the interplay of circadian processes in fear memory carries the potential to open new therapeutic avenues.
